# Replacement of branched-chain polyamine biosynthesis with thermospermine supports survival under both cold and heat stress in the hyperthermophilic archaeon *Thermococcus kodakarensis*

**DOI:** 10.1128/aem.00326-25

**Published:** 2025-05-28

**Authors:** Shinsuke Fujiwara, Riko Satake, Himari Aoki, Kaho Yamada, Yuri Ishii, Wakao Fukuda

**Affiliations:** 1 Department of Biosciences, Graduate School of Science and Technology, Kwansei Gakuin University 98370 , Sanda, Hyogo, Japan; 2 Department of Biosciences, School of Biological and Environmental Sciences, Kwansei Gakuin University 638727, Sanda, Hyogo, Japan; Colorado School of Mines, Golden, Colorado, USA

**Keywords:** archaea, branched chain polyamine, polyamine, thermophiles, *Thermococcus kodakarensis*

## Abstract

*N^4^*-bis(aminopropyl)spermidine (BCPA), a branched-chain polyamine, is uniquely found in bacterial and archaeal hyperthermophiles. In *Thermococcus kodakarensis*, BCPA is synthesized by BCPA synthase (BpsA), an aminopropyl transferase encoded by *bpsA*. This highly positively charged molecule is localized in both the nucleic acid and membrane fractions of *T. kodakarensis* cells. The *bpsA* deletion strain (DBP1), which lacks BCPA, failed to grow at 93°C and exhibited poor survival under repeated cold stress, indicating that BCPA is essential for membrane stability and function *in vivo*. Additionally, the expression of specific genes, including the cytoplasmic hydrogenase subunit *hyhL*, was absent in DBP1, suggesting a role for BCPA in gene regulation. To further investigate BCPA’s function, we replaced *bpsA* in *T. kodakarensis* with *speE* from the hyperthermophilic archaeon *Pyrobaculum calidifontis*, enabling the production of norspermine instead of BCPA. The resulting KPS strain accumulated thermospermine as its major polyamine. Growth at 93°C was partially restored in KPS, and cold-stress survival improved significantly. Additionally, KPS exhibited biosurfactant (sophorolipid) tolerance comparable to that of the parental *T. kodakarensis* strain KU216 under thermal conditions. Furthermore, *hyhL* expression was restored in KPS, as confirmed by immunoblotting with anti-HyhL antisera, suggesting that thermospermine can functionally compensate for BCPA. Notably, mutant DBP1 cells lacking both BCPA and thermospermine did not survive repeated cycles of cold and heat stress. This observation suggests that these polyamines play a crucial role in long-term survival, potentially facilitating hibernation-like states in natural environments where extreme temperature fluctuations occur.

**IMPORTANCE:**

At the hot springs of Kodakarajima Island, surrounded by cold ocean water, diverse hyperthermophiles, including *Thermococcus, Thermotoga,* and *Thermus* species, naturally produce branched-chain polyamines (BCPAs) via a unique aminopropyltransferase BpsA, in addition to spermidine. In *Pyrobaculum calidifontis*, the *Pc-*SpeE enzyme produces norspermine *in vivo*. However, when the *speE* gene from *P. calidifontis* is introduced into *Thermococcus kodakarensis*, the transformant (Δ*bpsA::Pc-speE*) produces thermospermine instead of norspermine. This shift suggests that the product specificity of *Pc-*SpeE is influenced by factors inherent to the host organism. Interestingly, thermospermine appears to functionally substitute for BCPA, potentially by forming BCPA-like structures with bent nitrogen atoms. This structural mimicry could contribute to cellular stability under both heat and cold stress, highlighting a potential mechanism for temperature and stress adaptation in *T. kodakarensis*. These findings further suggest that while BCPA and thermospermine are distinct, they may play similar roles in stress resilience.

## INTRODUCTION

**Fig 1:**
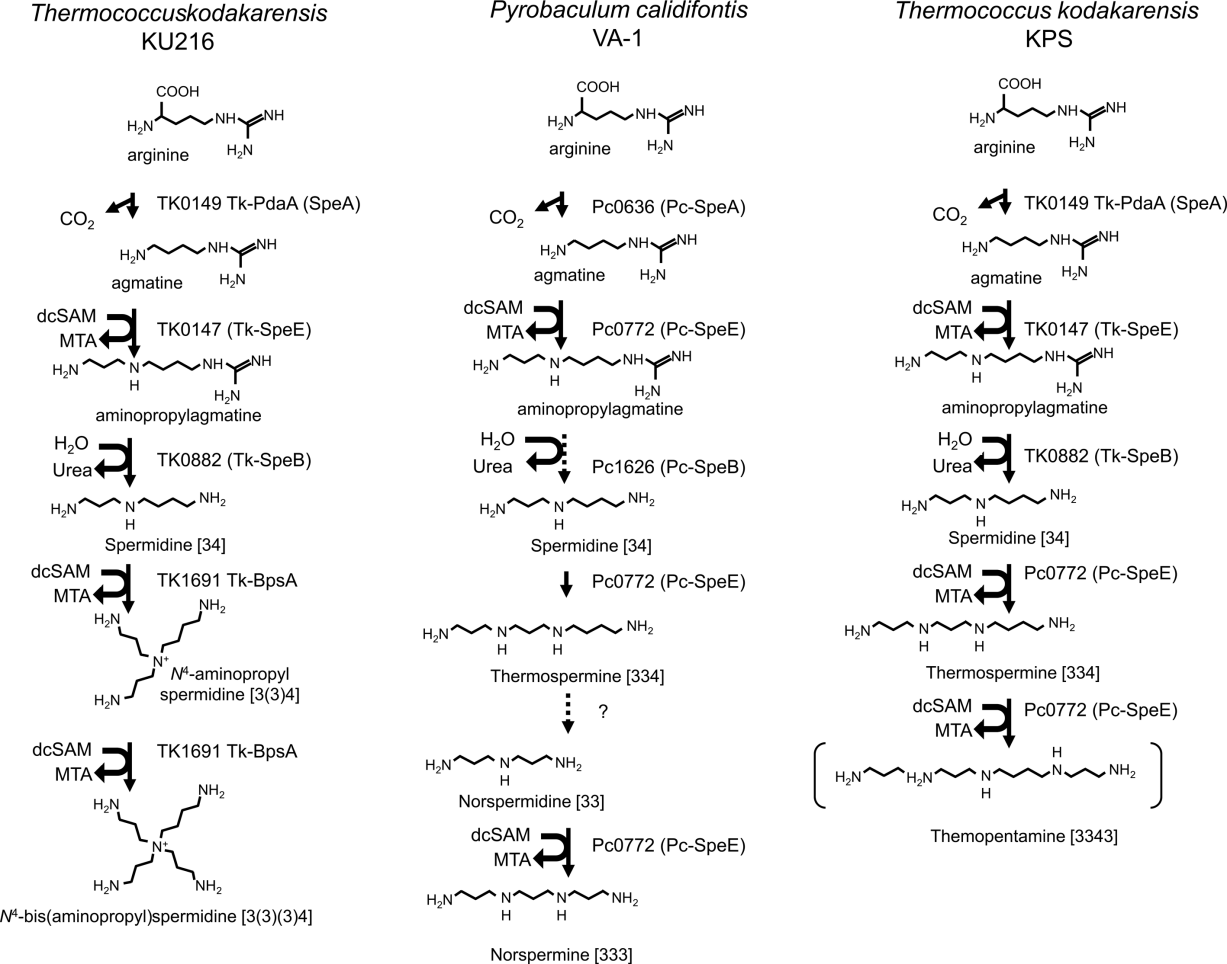
Predicted polyamine biosynthesis pathways in *Thermococcus kodakarensis* strain KU216, *Pyrobaculum calidifontis* VA1, and *Thermococcus kodakarensis* strain KPS. Solid black arrows indicate reactions supported by *in vitro* studies and gene knockout experiments. Dotted arrows represent reactions catalyzed by unidentified enzymes. Numbers in parentheses show the number of methylene (CH_2_) units between NH_2_ or NH groups. MTA, methylthioadenosine.

Polyamines are organic polycations with two or more primary amines, playing critical roles in numerous cellular processes such as transcription, translation, cell proliferation, differentiation, and adaptation to various stresses (1–3). Common polyamines like putrescine [4], spermidine [34], and spermine [343] (where numbers indicate methylene units between NH_2_ or NH groups) are synthesized in diverse organisms from arginine or ornithine, with aminopropyl groups from decarboxylated *S*-adenosylmethionine (dcSAM) added for larger polyamine synthesis. The hyperthermophilic archaeon *Thermococcus kodakarensis* synthesizes spermidine through the conversion of arginine to agmatine (a step catalyzed by arginine decarboxylase), aminopropylation of agmatine to *N*^1^-aminopropylagmatine (catalyzed by aminopropyltransferase), and hydrolysis of *N*^1^-aminopropylagmatine to spermidine by *N*^1^-aminopropylagmatine ureohydrolase (4). It is noteworthy that spermidine is synthesized without producing putrescine as an intermediate (Fig. 1). This unique synthesis pathway for spermidine via agmatine and *N*^1^-aminopropylagmatine was originally found in the thermophilic bacterium *Thermus thermophilus* (5). This pathway is considered a unique pathway only found in thermophiles. Branched-chain polyamines (BCPAs) are produced from spermidine in *T. kodakarensis*, essential for thermophile growth at elevated temperatures (6–8). BCPA synthase (BpsA), the enzyme responsible for BCPA biosynthesis, was first identified in the hyperthermophilic archaeon *T. kodakarensis*, which produces *N*^4^-bis(aminopropyl)spermidine [3(3)(3)4] via *N*^4^-aminopropylspermidine [3(3)4] for optimal growth at 85°C (4, 6). *N*⁴-aminopropylspermidine [3(3)4] is also a branched-chain polyamine. As we previously reported (9, 10), *N*⁴-bis(aminopropyl)spermidine [3(3)(3)4] is synthesized via *N*⁴-aminopropylspermidine [3(3)4] through a ping-pong reaction. However, the intermediate *N*⁴-aminopropylspermidine [3(3)4] is scarcely detected *in vivo* (6). Since the major branched-chain polyamine is *N*⁴-bis(aminopropyl)spermidine [3(3)(3)4], we refer to it simply as “BCPA” in the present study.

In *T. kodakarensis*, *N*^4^-bis(aminopropyl)spermidine [3(3)(3)4] is synthesized by the sequential addition of aminopropyl groups from dcSAM to spermidine [34] through BpsA’s bifunctional catalytic action (9). A similar catalytic mechanism was also found in BpsA from *T. thermophilus* (10). The absence of BpsA in the DBP1 strain of *T. kodakarensis* abolishes BCPA production, growth failure at 93°C, and the accumulation of spermidine during the lag phase (8). BCPAs have been shown to induce conformational changes in large DNA molecules, leading to mesh-like structures, unwinding, and nano-loops, suggesting a potential role in gene regulation at high temperatures (11, 12). Comparative transcriptomics and proteomics of DBP1 have revealed that BCPAs influence gene expression, with genes like HyhL absent in DBP1 (7).

Several hyperthermophiles, such as *Thermococcus*, *Thermotoga*, and *Thermus* species, were isolated from hot springs on Kodakarajima Island (13, 14), producing BCPAs likely for survival in fluctuating temperatures, as the island’s environment ranges from high temperatures in summer to near zero in winter. In contrast, proteoarchaeota like *Pyrobaculum calidifontis* strain VA1 from terrestrial hot springs lack BCPAs (15) and instead produce linear-chain polyamines like norspermidine [33] and norspermine [333] (16, 17). *P. calidifontis* also has an ortholog of arginine decarboxylase, contributing to polyamine biosynthesis along with aminopropyltransferase (*Pc*-SpeE), which favors norspermidine and norspermine production (17). *Pc*-SpeE’s substrate specificity suggests that thermospermine is formed from arginine via agmatine and spermidine. Norspermidine was shown to be synthesized from thermospermine through an unidentified polyamine oxidase (17) (Fig. 1).

In this study, we examined BCPA functions by replacing the *bpsA* gene in *T. kodakarensis* with the *speE* gene from *P. calidifontis*, which synthesizes thermospermine in place of BCPA, and assessed the resulting strain’s response to various stressors.

## RESULTS

### Unique polyamines in thermophiles and mutant construction to evaluate their roles

**Fig 2:**
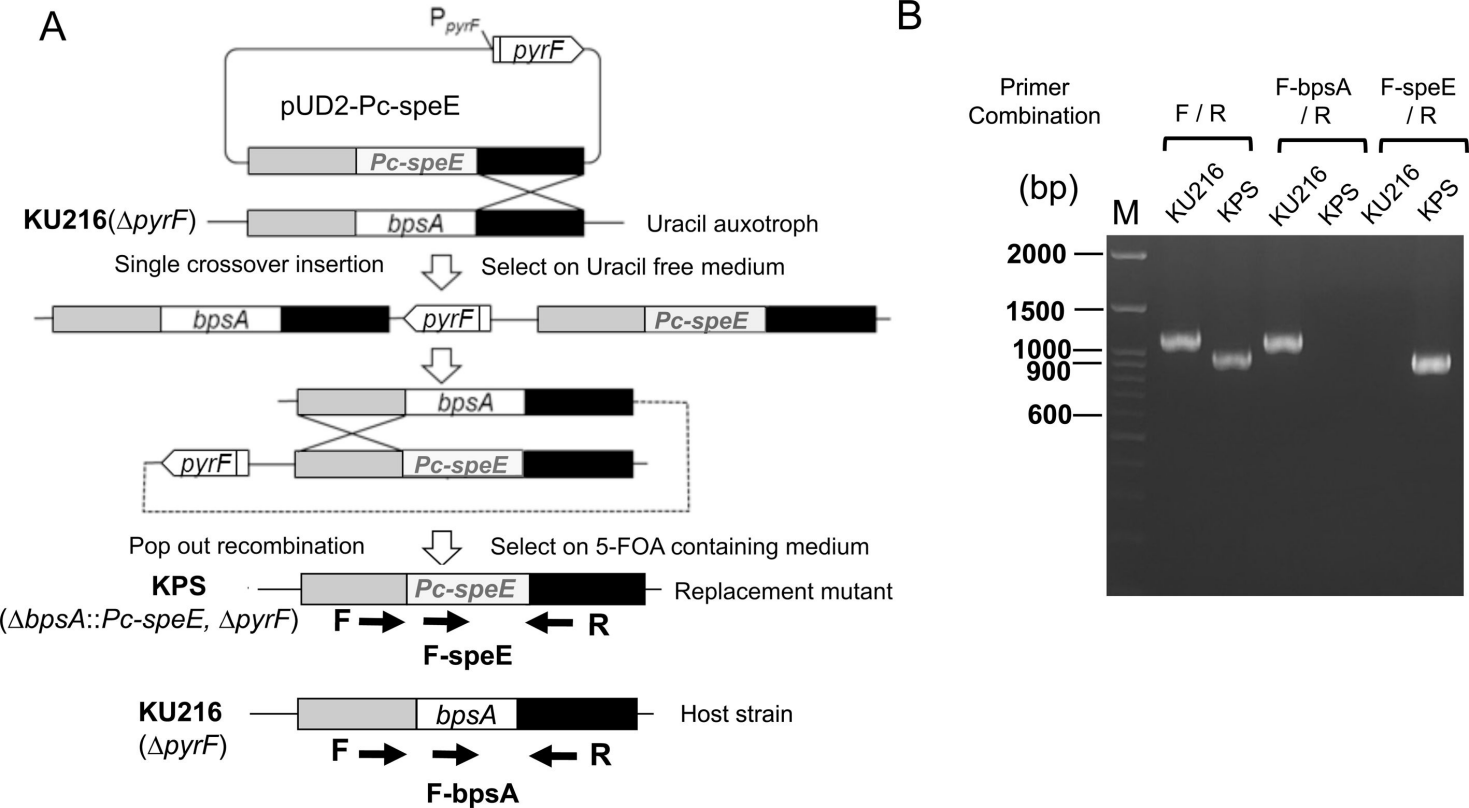
Targeted gene replacement via homologous recombination. (A) Schematic representation of KPS strain construction from *T. kodakarensis* KU216. Introduction of the replacement plasmid pUD2-Pc-speE into the parental strain KU216 resulted in the replacement of *Tk-bpsA* with *Pc-speE* from *P. calidifontis* through homologous recombination. Positions of primer-annealing sites used for PCR confirmation are indicated by arrows. (B) Agarose gel electrophoresis of PCR products from the genomic DNA of strains KU216 and KPS. PCR with primers F_pUD2-Pc-speE (indicated as “F”) and R_pUD2-Pc-speE (indicated as “R”) produced fragments of approximately 1,142 bp in KU216 and 956 bp in KPS from the 5′- and 3′-flanking regions of *bpsA*. Using primers F_speE and R_pUD2-Pc-speE (indicated as “R”), no PCR product was amplified in KU216, while a fragment of 933 bp was obtained from KPS. Using primers F_bpsA and R_pUD2-Pc-speE (indicated as “R”), no PCR product was amplified in KPS, while a fragment of 1,119 bp was obtained from KU216.

The branched-chain polyamine, *N*^4^-bis(aminopropyl)spermidine, is synthesized from spermidine by the aminopropyl transferase encoded by the *bpsA* gene. This gene has not been identified in mesophiles and is found exclusively in archaea and bacterial hyperthermophiles. Consequently, BCPA has been proposed to play a crucial role in adaptation to high-temperature environments. Interestingly, not all hyperthermophiles produce BCPA. For example, the archaeon *Pyrobaculum calidifontis* lacks cytoplasmic BCPA production. Instead, this hyperthermophile possesses the aminopropyl transferase SpeE, which synthesizes thermospermine from spermidine and norspermine from norspermidine (Fig. 1). To investigate the functional role of BCPA, we constructed a *Thermococcus kodakarensis* mutant strain in which the *bpsA* gene was replaced by *Pc-speE* from *P. calidifontis*. As described in Materials and Methods, this replacement mutant was generated through homologous recombination. The procedure is summarized in Fig. 2A. *T. kodakarensis* KU216 (*ΔpyrF*) was used as the parental wild-type strain in this study. KU216 is known to produce BCPA (6) and is useful for constructing deletion mutants via pop-in/pop-out homologous recombination using an uracil-deficient medium (18). Using plasmid pUD2-TK1691 (6) as a template, the upstream and downstream regions (approximately 1,000 bp each) flanking the *bpsA* coding sequence, as well as the plasmid pUD2 backbone, were amplified by inverse PCR. Separately, the *Pc-speE* region, including the 5′- and 3′-flanking regions of *bpsA*, was PCR amplified. These two DNA fragments were joined, resulting in the plasmid pUD2-Pc-speE. Transformation of strain KU216 cells with plasmid pUD2-Pc-speE was performed, and uracil-prototrophic colonies were selected on uracil-free ASW-AA medium. Transformants were further selected on ASW-YT medium containing 0.75% 5-fluoroorotic acid (5-FOA). Colonies were cultivated at 85°C, and genomic PCR using specific primers confirmed the replacement of *Tk-bpsA* with *Pc-speE*. The replacement was further validated by nucleotide sequence analysis. The resulting mutant strain was designated as KPS (*ΔbpsA::speE ΔpyrF*). As KPS cells were also grown at 85°C, investigating the polyamine content in the mutant became a compelling focus of our study. To this end, we evaluated the cytoplasmic polyamine profiles of KU216, DBP1, and KPS cells, aiming to uncover potential differences that may provide insights into the functional roles of polyamines in hyperthermophiles.

### Polyamine analysis

**Fig 3:**
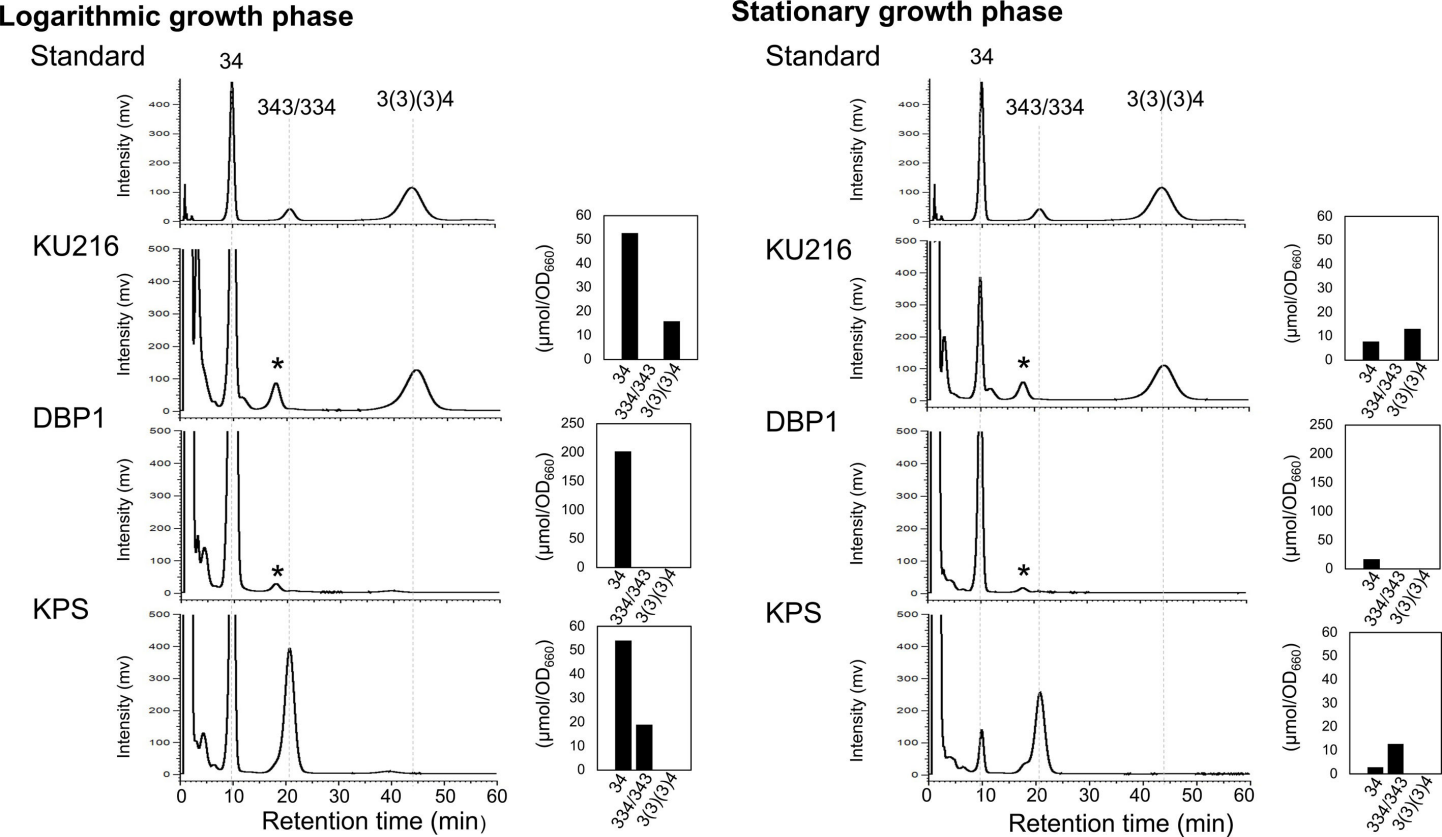
Polyamine composition of intracellular fractions in *T. kodakarensis* cells. *T. kodakarensis* strains KU216, DBP1, and KPS were cultivated separately in ASW-YT liquid medium supplemented with 0.5% (wt/vol) sodium pyruvate at 85°C until reaching the mid-logarithmic and stationary phases. Intracellular polyamines were extracted and analyzed by HPLC. An asterisk indicates an unknown peak. Also shown are graphic representations of polyamine profiles in each strain during the mid-logarithmic and stationary phases. 3(3)(3)4, *N*^4^-bis(aminopropyl)spermidine (BCPA); 34, spermidine; 343, spermine; 334, thermospermine.

**Fig 4:**
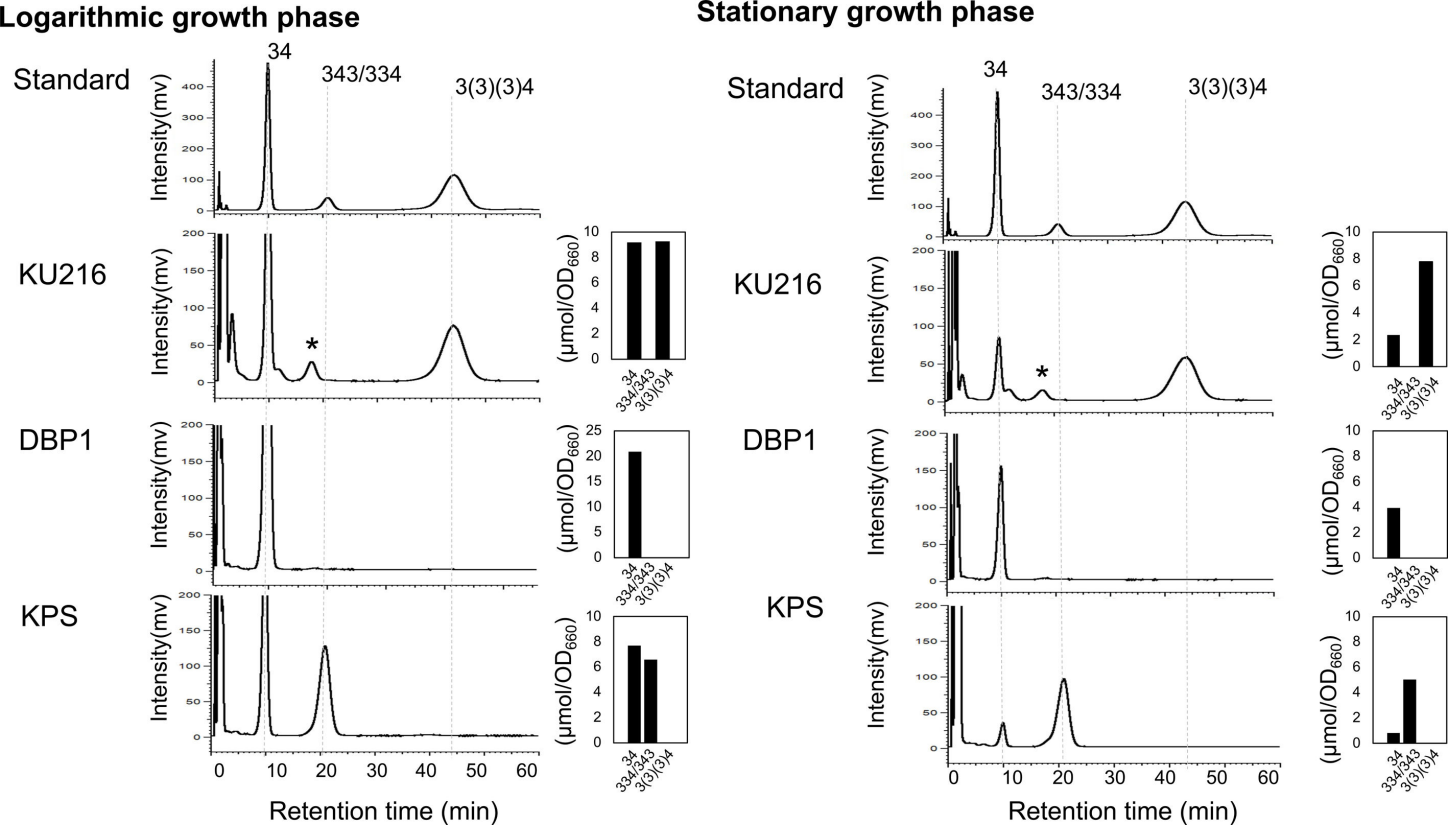
Polyamine composition of membrane fractions in *T. kodakarensis* cells. *T. kodakarensis* strains KU216, DBP1, and KPS were cultivated separately in ASW-YT liquid medium supplemented with 0.5% (wt/vol) sodium pyruvate at 85°C until reaching the mid-logarithmic and stationary phases. Polyamines of membrane fractions were collected and analyzed by HPLC. An asterisk indicates an unknown peak. Also shown are graphic representations of polyamine profiles in each strain during mid-logarithmic and stationary phases. 3(3)(3)4, *N*^4^-bis(aminopropyl)spermidine (BCPA); 34, spermidine; 343, spermine; 334, thermospermine.

*T. kodakarensis* strains KU216, DBP1, and KPS were cultivated separately in ASW-YT liquid medium supplemented with pyruvate at 85°C until reaching the mid-logarithmic and stationary growth phases. Intracellular polyamines were then extracted and analyzed by high-performance liquid chromatography (HPLC). Strain KU216 (*ΔpyrF*) served as the parental wild type, while strain DBP1 (*ΔpdaD ΔbpsA::pdaD ΔpyrF*) was used as a *bpsA* deletion mutant. As shown in Fig. 3, intracellular BCPA was detected in both the logarithmic and stationary phases of the wild-type strain KU216. In contrast, DBP1 accumulated an unusually high level of spermidine during the logarithmic phase, but spermidine levels decreased in the stationary phase. In the KPS strain, peaks corresponding to thermospermine/spermine and spermidine were observed (Fig. 3). The spermidine level in KPS was comparable to that of the wild-type KU216 (Fig. 3). Notably, the spermine [343] peak overlapped with that of thermospermine [334] in the HPLC analysis, making direct identification challenging. However, enzymatic analysis with recombinant *Pc*-SpeE confirmed that *Pc*-SpeE synthesizes thermospermine [334] from spermidine [34] (17). Based on this, the observed peak was interpreted as thermospermine [334]. Thermospermine [334] was identified as the major polyamine in KPS during the stationary phase, suggesting its critical role in survival under these conditions. Additionally, a minor peak corresponding to the long-chain polyamine thermopentamine [3343] was detected in trace amounts (data not shown), suggesting that thermopentamine [3343] may not play a significant functional role in KPS survival. We also examined the polyamine composition in membrane fractions of the three strains. In the stationary phase, KU216 and KPS cells contained higher levels of BCPA (*N*^4^-bis(aminopropyl)spermidine) [3(3)(3)4] and thermospermine [334] than spermidine (Fig. 4). This enrichment was more pronounced in the membrane fraction than in the intracellular fraction. BCPA and thermospermine may contribute to membrane stabilization in the stationary-phase cells of KU216 and KPS. These findings imply that these polyamines play a critical role in cellular survival under high-temperature conditions. To further investigate the physiological significance of these polyamines, we compared the growth of the mutant strains not only at 85°C but also at 93°C, which represents the upper growth temperature limit for the wild-type KU216.

### Comparison of growth profiles of mutant strains

**Fig 5:**
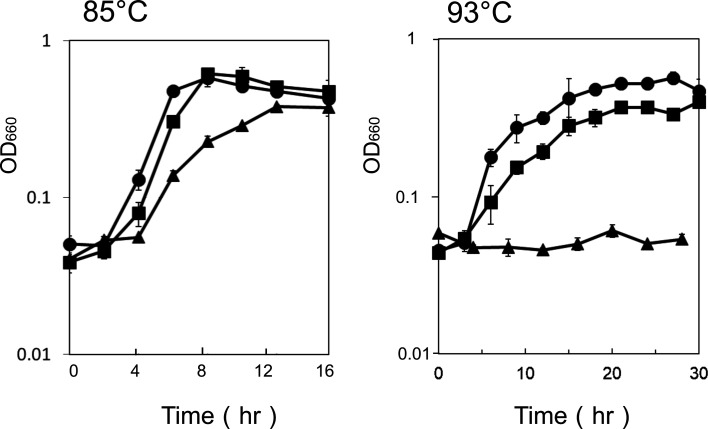
Growth phenotypes of KU216, DBP1, and KPS strains at different temperatures. Cultures of KU216 (circles), DBP1 (triangles), and KPS (squares) strains were grown at 85°C and 93°C in ASW-YT medium supplemented with 0.5% (wt/vol) sodium pyruvate.

Cells of *T. kodakarensis* strains KU216, DBP1, and KPS were inoculated into ASW-YT medium supplemented with 0.5% (wt/vol) sodium pyruvate and cultivated at 85°C and 93°C. As shown in Fig. 5, all strains were able to grow at 85°C. However, DBP1 exhibited a reduced growth rate, and its final cell yield was lower than those of KU216 and KPS. Notably, the growth rate and final cell yield of KPS were nearly identical to those of KU216. dcSAM also serves as a substrate for SpeE (*TK0147*) in *T. kodakarensis*, facilitating the production of aminopropyl agmatine. In DBP1, spermidine is abundantly accumulated, suggesting that dcSAM is efficiently utilized *in vivo* and does not accumulate to toxic levels. This observation suggests that thermospermine [334], the polyamine produced in KPS, plays a significant role in complementing the function of BCPA (*N*^4^-bis(aminopropyl)spermidine) [3(3)(3)4]. At 93°C, DBP1 cells were unable to grow, indicating the necessity of either BCPA or thermospermine [334] for high-temperature adaptation. In contrast, KPS cells showed slow growth, and their final cell yield was slightly lower than that of KU216.

### Cold-stress tolerance in *T. kodakarensis*

The natural habitat of *T. kodakarensis*, Kodakarajima Island, Japan, presents a unique environment where hot spring areas are closely associated with the ocean. During high tide, parts of the region are submerged under seawater. In winter, the area experiences near-freezing temperatures, making it a mixed thermal environment rather than exclusively high-temperature conditions. Various thermophiles, including species of *Thermus*, *Thermotoga*, and *Thermococcus*, have been isolated from this location and have served as sources of thermostable enzymes (13, 14, 19). Interestingly, all these organisms contain branched-chain polyamines, suggesting that BCPAs are essential not only for adaptation to high temperatures but also for survival under cold-stress conditions. This dual role of BCPAs is likely critical for hyperthermophiles inhabiting environments with fluctuating temperatures. In this study, we investigated the cold-stress tolerance of *T. kodakarensis* strains KU216, DBP1, and KPS. To mimic the natural environment, cells were subjected to repeated cycles of hot and cold stress under synthetic medium containing sea water components. Cultures were first grown at 85°C in ASW-YT-S medium for 17 hours and then reinoculated into oligotrophic ASW-AA-S medium for an additional 24 hours at 85°C. In the natural environment, available nutrients are limited. For these experiments, an oligotrophic medium was used to better mimic natural environmental conditions. ASW-AA-S, composed of amino acids, replaced the yeast extract and tryptone found in the richer ASW-YT-S medium. This simplified medium was chosen to reduce nutrient availability and enhance stress response analysis. Following growth, bottles containing the cultures were transferred to an ice-cold water bath for 30 minutes and then returned to an 85°C incubator for another 30 minutes. This hot-cold stress cycle was repeated four times. After each cycle, cells were plated on ASW-YT solid medium at various dilutions, and colony-forming units (CFUs) were determined by direct counting (Table S2).

**Fig 6:**
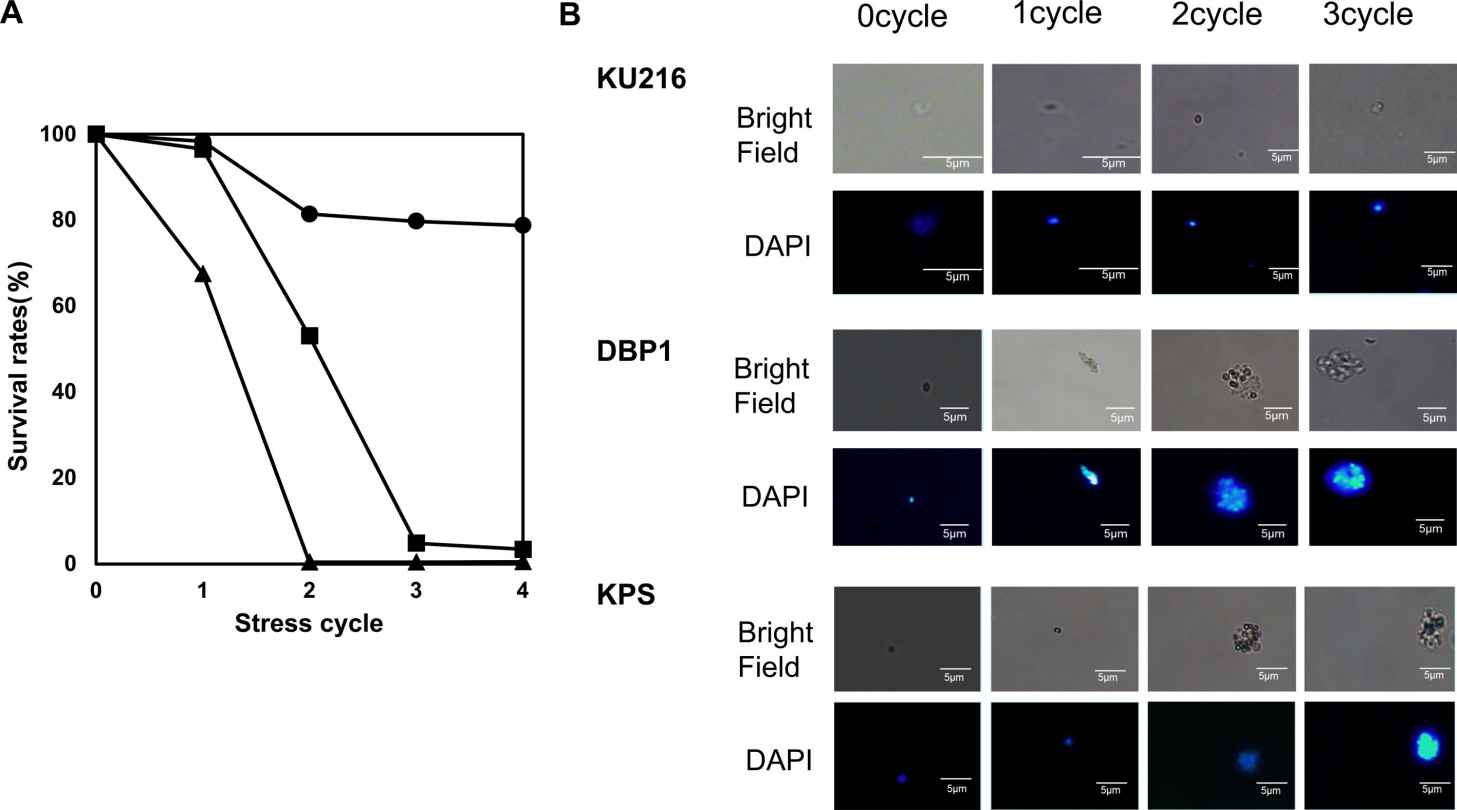
Cold-stress tolerance of *T. kodakarensis* strains KU216, DBP1, and KPS. (A) Cell survival rates after varying cycles of cold-stress treatment. Cells cultivated at 85°C in ASW-AA-S medium bottles were transferred to an ice-cold water bath and kept for 30 minutes, then returned to an 85°C incubator for 30 minutes. This cycle was repeated four times. After cold stress, cells were plated on ASW-YT solid medium at various dilutions, and CFUs were counted directly. The survival rate was calculated as CFU relative to non-cold-stressed cells, defined as 100%. Circles, KU216; triangles, DBP1; squares, KPS. (B) Microscopic observation of cells after cold stress. Cells from strains KU216, DBP1, and KPS were observed by microscopy following repeated cold-stress treatment. Brightfield and fluorescence imaging were conducted on cells stained with 4′,6-diamidino-2-phenylindole dihydrochloride (DAPI). Fluorescence signals were detected at an emission wavelength of 440 nm with an excitation wavelength of 360 nm.

As shown in Fig. 6A, approximately 80% of wild-type KU216 cells survived after four cycles of cold stress. In contrast, survival of DBP1 cells decreased to 70% after the first cold stress cycle and dropped to nearly 0% following the second cycle, highlighting the crucial role of BCPAs in cold-stress survival. For KPS cells, around 50% survived up to the second cold-stress cycle, but a significant decline was observed after the third cycle. These results indicate that BCPAs are essential for the cold-stress tolerance of *T. kodakarensis*, while thermospermine provides partial but insufficient rescue for complete survival.

### Microscopic observation of cold-stressed cells of *T. kodakarensis* strains

Cells from *T. kodakarensis* strains KU216, DBP1, and KPS were examined following repeated exposure to cold stress. Brightfield and fluorescence microscopy were employed, with cells stained using 4′,6-diamidino-2-phenylindole dihydrochloride (DAPI). Fluorescence was detected at an excitation wavelength of 360 nm and an emission wavelength of 440 nm. As shown in Fig. 6B, strain KU216 exhibited primarily single-cell morphology under both brightfield and fluorescence microscopy. In contrast, strain DBP1 displayed significant cell aggregation and clumping, a phenomenon particularly evident in DAPI-stained images. For strain KPS, cells remained as single entities following the first cold stress treatment but exhibited marked aggregation after three cycles of cold stress, similar to the pattern observed in DBP1. These findings suggest that BCPA or thermospermine may influence interactions with membrane components, thereby playing a role in maintaining cell membrane stability under cold stress conditions. To further investigate this, the subsequent section explores the impact of surfactants on cell viability in *T. kodakarensis* strains.

### Effect of surfactant on cell growth of *T. kodakarensis* strains

**Fig 7:**
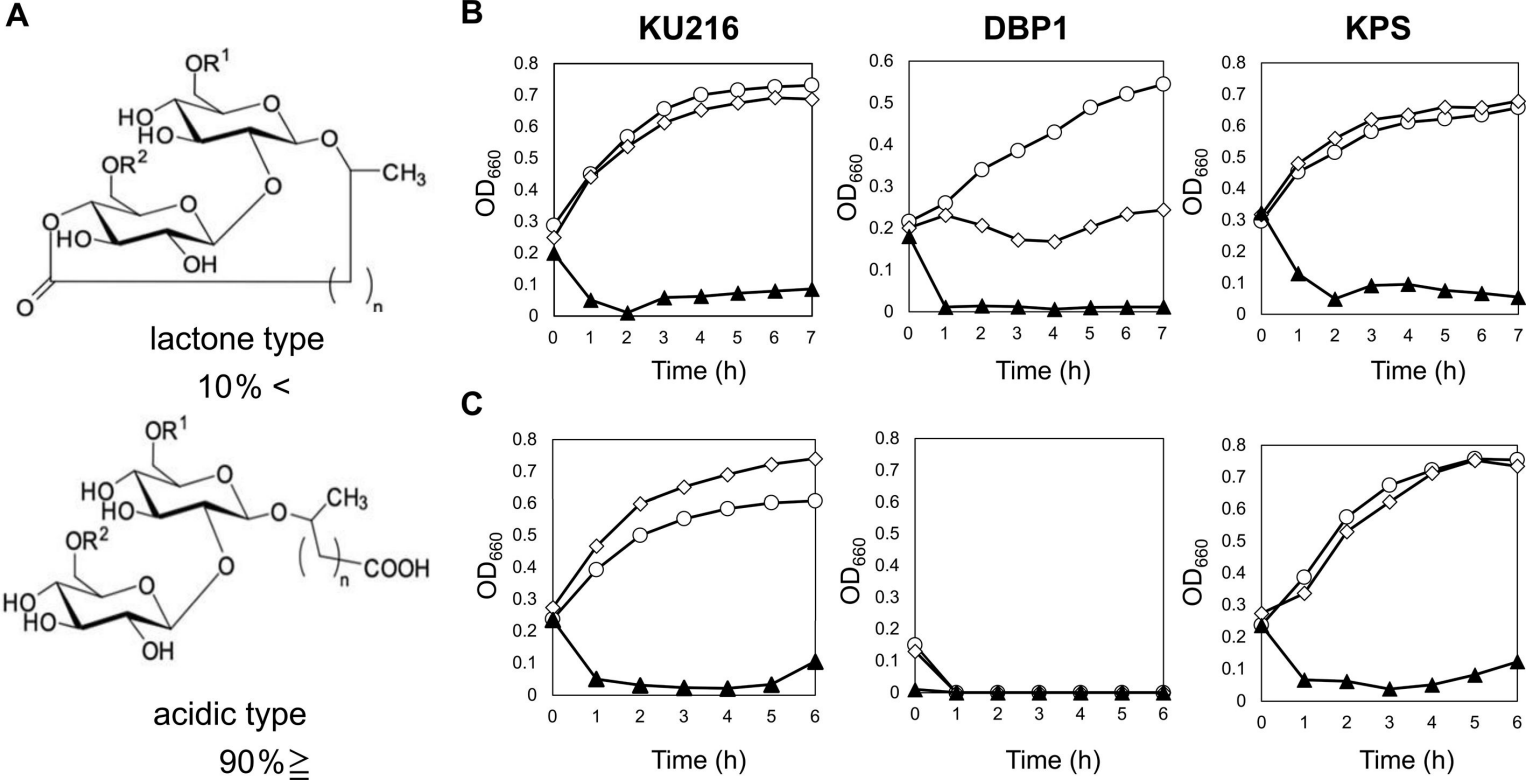
Effect of sophorolipid on the growth of *T. kodakarensis* strains. (A) Chemical structure of sophorolipid. R^1^ and R^2^ indicate hydrogen or acetyl groups. (B) Cell growth at 85°C. (C) Cell growth at 90°C. Cells were initially cultivated overnight at 85°C in ASW-YT-S medium. A 1% inoculum was transferred and cultured for 24 hours at 85°C and 90°C in ASW-YT medium supplemented with 0.5% (wt/vol) sodium pyruvate. These cultures were then inoculated into fresh ASW-YT pyruvate medium containing sophorolipid at the indicated concentrations and grown at 85°C (B) and 90°C (C). Growth was monitored by measuring OD_660_. Symbols represent sophorolipid concentrations: 〇 (open circle), 0 µg/mL; ◇ (open diamond), 10 µg/mL; and ▲ (closed triangle), 50 µg/mL.

The DBP1 strain exhibited heightened sensitivity to cold stress, suggesting that the absence of BCPA in the cytoplasm affects membrane components. BCPA, a highly positively charged polyamine, is hypothesized to interact with cytoplasmic membrane lipids, contributing to membrane stability. Previous proteomic analysis indicated that TK1577, a putative ABC-type multidrug transporter membrane protein in *T. kodakarensis* KU216, was undetectable in DBP1 cells (7). These findings imply that the loss of BCPA synthesis renders mutant strains more sensitive to surfactants. Although hyperthermophiles are unlikely to encounter synthetic surfactants in their natural environments, they may encounter biosurfactants. In this study, we investigated the impact of the thermostable biosurfactant sophorolipid, which is naturally produced by the yeast *Starmerella bombicola*. Sophorolipid consists of two structural forms: approximately 10% lactone type and 90% acid type (Fig. 7A). The growth of *T. kodakarensis* strains KU216, DBP1, and KPS was evaluated in ASW-YT medium supplemented with varying concentrations of sophorolipid (Fig. 7B and C). At 85°C, KU216 and KPS cells exhibited tolerance to sophorolipid at concentrations up to 10 µg/mL, whereas DBP1 cells failed to grow at this concentration. OD_660_ of all strains in ASW-YT medium containing 50 µg/mL sophorolipid decreased as shown in Fig. 7. Cell lysis of all strains was confirmed by microscopic observation. At 90°C, KU216 and KPS cells maintained tolerance at concentrations up to 10 µg/mL sophorolipid, while DBP1 cells failed to grow even in the absence of sophorolipid. These findings suggest that BCPA in strain KU216 and thermospermine [334] in strain KPS contribute to membrane stability, either directly or indirectly, thereby enhancing tolerance to the biosurfactant sophorolipid under thermal conditions.

### HyhL expression in *T. kodakarensis* strains

**Fig 8:**
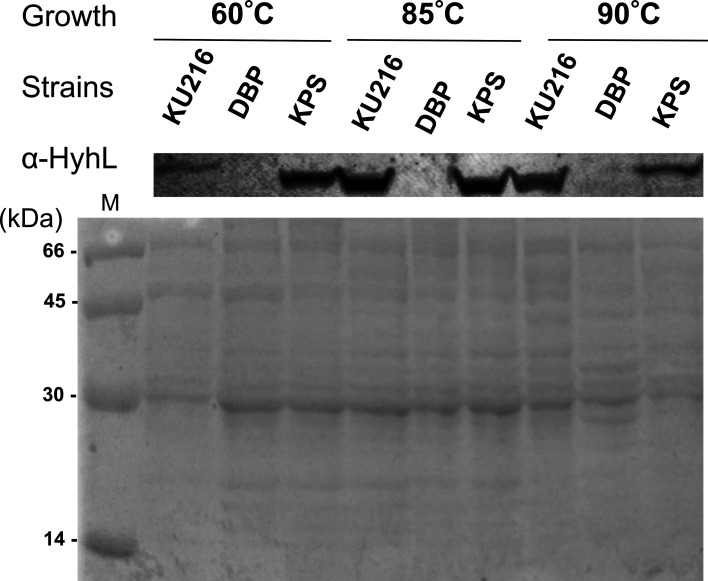
Intracellular levels of HyhL in *T. kodakarensis* strains grown at different temperatures. Cell extracts (10 µg) from *T. kodakarensis* strains KU216, DBP1, and KPS grown at 60°C, 85°C, or 90°C were analyzed by immunoblotting. Samples were separated by polyacrylamide gel electrophoresis and transferred onto a polyvinylidene difluoride membrane. HyhL was detected using anti-HyhL antisera.

Previous comparative proteomic analyses revealed that the HyhL subunit of the cytoplasmic hydrogenase complex was detected in strain KU216 but not in DBP1, despite the presence of *hyhL* mRNA in both strains (7). These findings suggest that *hyhL* expression in *T. kodakarensi*s is regulated at a post-transcriptional level in a BCPA-dependent manner. This dependence on BCPA for *hyhL* expression was observed at 60°C, 85°C, and 90°C. To evaluate *hyhL* expression in strain KPS, cells were cultivated at the three temperatures mentioned above, and protein expression was analyzed via immunoblotting using anti-HyhL antisera. As shown in Fig. 8, HyhL expression was detected in KPS cells under all tested conditions, indicating that thermospermine in KPS complements the function of BCPA in the cytoplasm.

### Evaluation of polyamine effects on *hyhL* expression using an *in vitro* cell-free system

**Fig 9:**
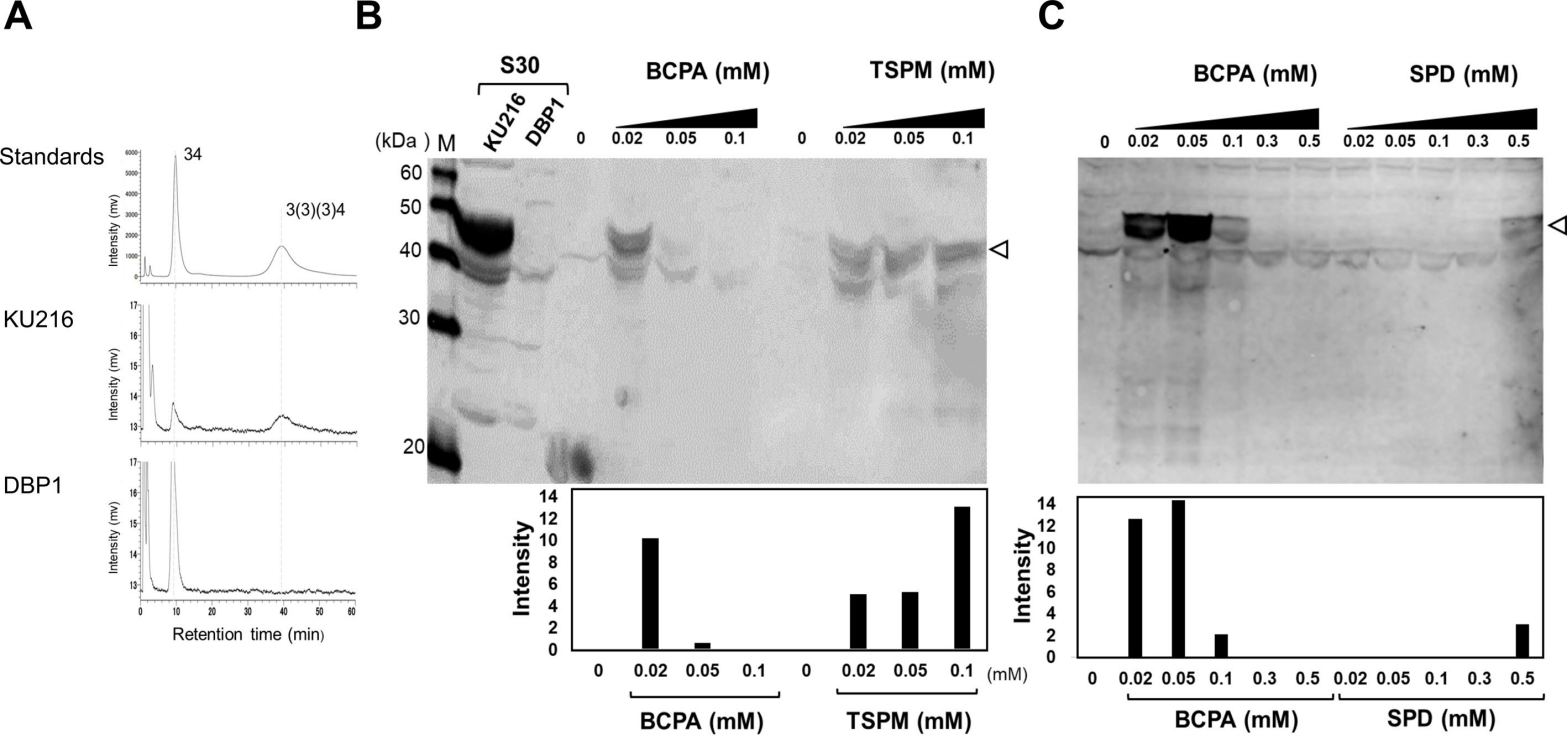
Cell-free synthesis of HyhL protein using *T. kodakarensis* S30 extract. (A) Polyamine composition of the S30 extract. (B) Detection of HyhL in S30 extracts from KU216 and DBP1 cells, as well as *in vitro*-synthesized HyhL in the presence of BCPA and thermospermine. (C) *In vitro* synthesis of HyhL in the presence of BCPA and spermidine. For *in vitro* translation, reaction mixtures containing 1 µg of *hyhL* mRNA were incubated at 65°C for 60 minutes with or without the polyamines BCPA, thermospermine, and spermidine. Following translation, reaction products were analyzed by SDS-PAGE (10% acrylamide), and HyhL was detected via immunoblotting using anti-HyhL antisera. BCPA, *N*^4^-bis(aminopropyl)spermidine [3(3)(3)4]; SPD, spermidine [34]; TSPM, thermospermine [334].

To investigate the polyamine dependency of *hyhL* expression observed in KU216 and KPS strains, *in vitro* translation experiments were conducted. For the *in vitro* experiment, the supernatant fraction obtained after centrifugation at 30,000 × *g* (S30), which removes cell debris while keeping ribosomes, tRNA, enzymes, and other factors needed for translation, was utilized. S30 extracts were prepared from DBP1 cells cultivated at 90°C. As shown in Fig. 9A, HyhL protein was detected in the S30 extract from KU216 cells but was undetectable in the extract from DBP1 cells, as determined by Western blot analysis using anti-HyhL antisera. For *in vitro* translation, *hyhL* mRNA was synthesized using the T7 RNA polymerase system (T7 RiboMAX Express Large Scale RNA Production System, Promega). One microgram of synthesized *hyhL* mRNA was incubated with the S30 extract at 65°C for 60 minutes in the presence of varying concentrations of spermidine, thermospermine, and BCPA. Following translation, reaction mixtures were analyzed by SDS-PAGE, and HyhL protein levels were assessed via Western blotting using an anti-HyhL antibody. As shown in Fig. 9B, the addition of 0.02–0.05 mM BCPA resulted in a clear detection of HyhL, whereas only a faint signal was observed at 0.1 mM BCPA. Similarly, thermospermine [334] induced HyhL expression starting at 0.02 mM, with consistent expression observed at higher concentrations. The concentration dependency varied between BCPA and thermospermine. BCPA induced HyhL expression at the lowest concentration, but its effect diminished at higher concentrations, suggesting a finely tuned regulatory mechanism *in vivo*. In contrast, thermospermine [334] supported HyhL expression across a broader concentration range, without the sharp dose-dependent response observed for BCPA. The effect of spermidine addition on HyhL expression was also examined (Fig. 9C). No detectable HyhL signal was observed up to 0.3 mM spermidine, and a weak signal was detected at 0.5 mM. However, concentrations above 0.5 mM are likely abnormally high and may not naturally occur in the cytoplasm. The requirement for higher spermidine concentrations suggests that its role in *hyhL* induction is limited under physiological conditions. These *in vitro* findings indicate that the regulatory mechanism underlying BCPA-dependent *hyhL* induction differs from that of thermospermine [334]. Furthermore, the results highlight the precise regulatory role of BCPA in controlling *hyhL* expression, reinforcing its physiological significance in *T. kodakarensis*.

## DISCUSSION

*Thermococcus kodakarensis* was originally isolated from hot springs on Kodakarajima Island. These hot springs also harbor other hyperthermophilic genera, including *Thermus* species and *Thermotoga* species, all of which uniquely produce branched-chain polyamines—polyamines found exclusively in hyperthermophiles. BCPA is synthesized via the aminopropyltransferase activity of BpsA through a ping-pong mechanism (9, 10). Deletion of the *bpsA* gene in *T. kodakarensis* results in a mutant strain (DBP1) that cannot grow at 93°C, a temperature tolerated by the wild-type KU216 strain. This finding highlights the importance of BCPA for growth at high temperatures.

However, the hot spring environment of Kodakarajima is not consistently at high temperatures. Many of these springs form tide pools along the coastline, which are submerged at high tide and exposed to air at low tide. During winter, ambient temperatures in this region can occasionally drop to 0°C. Hyperthermophiles must adapt to such fluctuating conditions to survive. We hypothesize that *T. kodakarensis* has persisted since its emergence due to its ability to withstand low-temperature stress, with BCPA playing a crucial role in stabilizing the cell membrane under such conditions. Supporting this hypothesis, DBP1 cells lacking BCPA exhibit heightened sensitivity to biosurfactants, suggesting reduced membrane stability compared to BCPA-containing cells. The increased susceptibility of DBP1 cells to both low-temperature and biosurfactant-induced stress observed in this study implies that *T. kodakarensis* mutants lacking BCPA synthesis would struggle to survive in natural environments. This could explain why no hyperthermophiles deficient in BCPA production have been isolated from Kodakarajima’s hot springs. Beyond *T. kodakarensis*, many species of *Thermococcus* and *Pyrococcus*, which are phylogenetically closely related hyperthermophiles, have been isolated from diverse high-temperature environments worldwide, particularly from marine and coastal regions. Like *T. kodakarensis* in Kodakarajima, these organisms likely encounter cold stress in their native habitats. Notably, all sequenced *Thermococcus* and *Pyrococcus* genomes contain a *bpsA* ortholog, suggesting that BCPA is a conserved and essential polyamine for survival under both high-temperature and cold-stress conditions. KU216 cells exhibited higher levels of BCPA [3(3)(3)4] than spermidine [34] during the stationary phase, with this enrichment being more pronounced in the membrane fraction than in the intracellular fraction. This observation suggests that BCPA plays a crucial role in membrane stabilization, not only during the stationary phase but also during prolonged survival (hibernation) in natural environments.

In the present study, we constructed the mutant strain KPS by replacing the *bpsA* gene with *speE* from *Pyrobaculum calidifontis*. Unlike *T. kodakarensis*, *P. calidifontis* does not produce BCPA, as it lacks the *bpsA* gene. Instead, *P. calidifontis* utilizes a unique metabolic pathway to produce norspermine [333]. Recombinant *Pc*-SpeE synthesizes both thermospermine [334] and spermine [343] from spermidine [34] and dcSAM. *Pc*-SpeE exhibits high enzymatic activity with aminopropylagmatine and norspermidine as substrates, but low affinity for putrescine, which is not stably bound in its active site. In *P. calidifontis*, norspermine [333] is primarily produced via thermospermine [334] through oxidative degradation, followed by further conversion by *Pc-*SpeE. Interestingly, norspermine [333] is relatively abundant in *P. calidifontis* cytoplasm compared to BCPA levels in *T. kodakarensis* (17). Although DBP1 cells lack BCPA, they exhibit limited growth, potentially due to the compensatory effects of excess spermidine [34] in the cytoplasm. In the KPS strain, thermospermine [334] was likely synthesized from spermidine [34] and appeared to compensate for the absence of BCPA. It is plausible that thermospermine [334] mimics BCPA’s functional role by forming BCPA-like tertiary structures through bending at nitrogen atoms. This structural mimicry may contribute to the stabilization of cellular components and adaptation to environmental stressors.

Expression of the *hyhL* gene has been shown to occur in the presence of BCPA (7). Notably, *hyhL* mRNA levels remain consistent between KU216 and DBP1 strains, suggesting that BCPA promotes *hyhL* translation at the post-transcriptional level. This hypothesis was supported by S30 cell-free translation experiments, where *hyhL* expression was promoted by the addition of 0.02–0.05 mM BCPA but was not observed at 0.1 mM. These findings indicate that BCPA regulates *hyhL* translation in a concentration-dependent manner. In the wild-type KU216 strain, the HyhL protein is consistently expressed under all tested conditions across all evaluated temperatures (Fig. 8). This suggests that cytoplasmic BCPA levels in KU216 are tightly regulated, preventing accumulation that could inhibit *hyhL* translation.

Interestingly, *hyhL* expression was also observed in the KPS strain (Fig. 8), indicating that intracellularly synthesized thermospermine [334] can functionally replace BCPA. *In vitro* experiments further confirmed *hyhL* translation in the presence of thermospermine [334] at concentrations ranging from 0.02 to 0.1 mM (Fig. 9). However, unlike BCPA, thermospermine [334] did not promote *hyhL* expression in a concentration-dependent manner. This suggests that while thermospermine can substitute for BCPA, its regulatory mechanism may lack the precision observed with BCPA. In DBP1 cells, HyhL was not detected at any cultivation temperature (Fig. 8), consistent with the absence of BCPA. However, *in vitro* translation experiments demonstrated that *hyhL* expression could be induced by 1.0 mM spermidine [34]. Such high concentrations of spermidine likely provide a compensatory effect, albeit under artificial conditions, as this concentration probably far exceeds physiological levels typically found in the cytoplasm during natural cultivation.

The precise mechanisms by which BCPA [3(3)(3)4] and thermospermine [334] regulate *hyhL* translation remain unclear. While thermospermine [334] can partially mimic BCPA’s role, it does not appear to achieve the same level of translational control, highlighting the unique properties of BCPA in facilitating *hyhL* expression.

## MATERIALS AND METHODS

### Microorganisms and media

*Thermococcus kodakarensis* KU216 (Δ*pyrF*) (20) was used as the parental host strain for mutant construction. Strain KU216 and its derivatives (Table S1) were cultivated anaerobically in a nutrient-rich medium, ASW-YT (21), supplemented with 2.0 g L⁻¹ elemental sulfur (ASW-YT-S), ASW-YT with 0.5% (wt/vol) sodium pyruvate (ASW-YT pyruvate), or in a synthetic medium (ASW-AA) (21) containing 0.8× artificial seawater (ASW), amino acids, and elemental sulfur. For solid medium, 1% Gelrite (Wako, Osaka, Japan) and 2 mL L⁻¹ polysulfide solution (10 g Na_2_S·9H_2_O and 3 g sulfur flowers dissolved in 15 mL H_2_O) were added. When 5-FOA was required for selection, ASW-YT medium containing 0.75% 5-FOA was used. *Escherichia coli* strain DH5α cells were cultivated at 37°C in Lysogeny Broth medium. Ampicillin (50 µg mL⁻¹) was added to the medium when required for selection.

### Construction of gene replacement mutant KPS

The principles underlying specific gene disruption in *T. kodakarensis* have been previously described (18, 20). The procedure used here is summarized in Fig. 2A. Using plasmid pUD2-TK1691 as a template, upstream and downstream regions (approximately 1,000 bp each) and the entire pUD2 region were amplified by inverse PCR with primers Revec_Tk1691_updown_Inf_Fw and Revec_Tk1691_updown_Inf_Rv. Additionally, the *Pc-speE* region, along with the 5′- and 3′-flanking regions of *bpsA*, was PCR amplified using primers Ins_PcSpeE_Tk1691_updown_Inf_Fw and Ins_PcSpeE_Tk1691_updown_Inf_Rv. The two resulting PCR-amplified DNA fragments were joined using the In-Fusion Cloning Kit (Takara Bio Co., Shiga, Japan), and the resulting plasmid was designated as pUD2-*Pc-speE*. The nucleotide sequence of pUD2-*Pc-speE* was confirmed using primers PespeE_seq_Fw, PespeE_seq_Md, and PespeE_seq_Rv. Plasmid pUD2-*Pc-speE* was used to transform strain KU216 cells, and colonies were selected on uracil-free ASW-AA medium. Transformants were further selected on ASW-YT medium containing 0.75% 5-FOA. Successful gene replacement was confirmed by nucleotide sequencing.

### Polyamine analysis

*T. kodakarensis* strains were cultivated in ASW-YT medium supplemented with 0.5% (wt/vol) sodium pyruvate at 85°C until reaching the logarithmic and stationary phases, after which they were harvested. Cells were disrupted by sonication in 50 mM Tris-HCl (pH 8.0), and the supernatant was collected as the intracellular fraction. Trichloroacetic acid (TCA) was added to a final concentration of 10% (vol/vol), and the solution was centrifuged at 20,400 × *g* for 15 minutes. The resulting supernatant was filtered through a 0.45 µm pore-size filter (SEPARA syringeless filter vial, GVS S.p.A., Bologna, Italy). A 100 µL aliquot of the filtered supernatant was analyzed by high-performance liquid chromatography using a CK-10S cation-exchange column (6.0 mm [i.d.] × 50 mm; GL Science, Tokyo, Japan). The column was equilibrated with a modified elution buffer (100 mM potassium citrate monohydrate, 2.0 M KCl, 650 mM 2-propanol, and 2.4 mM Brij35; pH 3.2, adjusted by adding 65 mL of 3 M HCl per liter) at a flow rate of 1.0 mL minute⁻¹ at 70°C. The elute was automatically mixed with a detection buffer composed of 400 mM boric acid, 400 mM NaOH, 4.9 mM Brij35, 7.5 mM *o*-phthalaldehyde, 171 mM ethanol, and 28 mM 2-mercaptoethanol at a flow rate of 0.5 mL minute⁻¹ at 70°C. Polyamines were detected using a fluorescence detector (RF-10AXL; Shimadzu, Kyoto, Japan). After cell disruption and centrifugation, the precipitate was collected as the membrane fraction. To solubilize the membrane fraction, TCA was added to a final concentration of 10% (vol/vol), and the mixture was centrifuged at 20,400 × *g* for 15 minutes. The resulting supernatant was filtered through a 0.45 µm pore-size filter, and a 100 µL aliquot was analyzed by HPLC as described above.

### Microscopic observation

Cells from strains KU216, DBP1, and KPS were observed by microscopy. Brightfield and fluorescence imaging were performed on cells stained with DAPI. Cell suspensions were mixed with an equal volume of DAPI solution (1 mg/mL). Fluorescence signals were detected at an emission wavelength of 440 nm with an excitation wavelength of 360 nm.

### Viable cell counts after repeated cold stress

*T. kodakarensis* cells were cultivated at 85°C in ASW-YT-S medium in bottles for 17 hours and reinoculated into ASW-AA-S medium for further cultivation at 85°C for 24 hours. Bottles containing grown cells were transferred to an ice-cold water bath and incubated for 30 minutes. Following cold treatment, the bottles were returned to an 85°C incubator for 30 minutes. This cycle was repeated four times. After each cold stress cycle, cells were plated on ASW-YT solid medium at various dilution rates. CFUs were determined by direct colony counting.

### Tolerance to sophorolipid

Sophorolipid was kindly provided by Saraya Co. Ltd. (Osaka, Japan). Cells were pre-cultivated at 85°C in ASW-YT liquid medium supplemented with 0.5% (wt/vol) sodium pyruvate for 17 hours. Then, 0.1 mL of the preculture was inoculated into 10 mL of ASW-YT pyruvate liquid medium in the presence of sophorolipid at various concentrations and further cultivated at 85°C and 90°C. Growth was monitored by measuring optical density at 660 nm.

### Western blot analysis

Cell extracts (10 µg) from *T. kodakarensis* strains cultivated at 60°C, 85°C, or 90°C were subjected to electrophoresis in 12% polyacrylamide gels containing 0.1% sodium dodecyl sulfate and transferred onto polyvinylidene difluoride membranes (Bio-Rad, Tokyo, Japan). Membranes were blocked with 5% skimmed milk and incubated with rabbit anti-HyhL antisera (a generous gift from Dr. Tamotsu Kanai, Kyoto University) at a 1:40,000 dilution (22). Immunocomplexes were detected using Alexa Fluor 700-conjugated goat anti-rabbit IgG (H + L) secondary antibody (Thermo Fisher Scientific). Signals were visualized using an Odyssey infrared imaging system (LI-COR Biosciences, Lincoln, NE, USA).

### Preparation of *T. kodakarensis* S30 extract

S30 extract was prepared from 1.6 L of *T. kodakarensis* DBP1 cells cultivated at 90°C in ASW-YT medium supplemented with 0.5% (wt/vol) sodium pyruvate under anaerobic conditions, following optimized procedures (23). Cells were washed once with S30 buffer (10 mM Tris-acetate, pH 7.4, 1 mM dithiothreitol, 1.4 mM magnesium acetate, and 6.0 mM potassium acetate) supplemented with 0.05% (vol/vol) 2-mercaptoethanol and collected by centrifugation at 3,000 × *g* for 15 minutes. Cells were resuspended in S30 buffer (1.27 mL per gram of wet cells) and disrupted by sonication. Dithiothreitol was added to the lysate to a final concentration of 1 mM. The lysate was centrifuged at 30,000 × *g* for 30 minutes at 4°C, and the upper four-fifths of the supernatant was collected. This process was repeated, and the upper four-fifths of the supernatant was collected again. For each 1 mL of supernatant, dialysis was performed three times (45 minutes each) against 40 volumes of S30 buffer using 5000 MWCO dialysis tubes. After final centrifugation at 4,000 × *g* for 10 minutes, the supernatant was used as the S30 extract. Protein concentrations were measured by the Bradford assay using BSA as a standard, and the S30 extract was stored at −80°C until use.

### Cell-free translation

*In vitro* translation was performed based on established conditions (23). Plasmid pTRC1 (23) was kindly provided by Dr. Tamotsu Kanai (Kyoto University). Using primers hyhL_Fw and hyhL_Rv, a 1,538 bp DNA fragment containing the *hyhL* region (including a 200 bp upstream region) was amplified using *T. kodakarensis* genomic DNA as a template. The T7 promoter region of plasmid pTRC1 (excluding the *chiA* gene) was amplified using primers pTRC11_Fw and pTRC11_RV. The two fragments were connected using an Infusion Kit (Takara Bio), and the resulting plasmid was designated as pTRC11. Plasmid pTRC11 was digested with EcoRI, and the linearized DNA was used for *in vitro* RNA synthesis with the T7 RiboMAX Express Large Scale RNA Production System (Promega, Tokyo, Japan). Protein synthesis reactions were carried out in a 30 µL mixture containing 1 µg of mRNA, *T. kodakarensis* S30 extract (5 mg/mL), magnesium acetate (4.0 mM), potassium acetate (250 mM), ammonium acetate (80 mM), Tris-acetate (56 mM, pH 8.2), ATP (3.0 mM), GTP (1.5 mM), CTP (1.5 mM), UTP (1.5 mM), potassium phosphoenolpyruvate (10 mM), polyethylene glycol 8000 (2%, wt/vol), and 20 amino acids (2.0 mM each). Various concentrations of polyamines (spermidine, thermospermine, and *N*^4^-bis(aminopropyl)spermidine) were added. The reaction mixtures were incubated at 65°C for 60 minutes. After translation, reaction mixtures (10 µL) were analyzed by SDS-PAGE (10% acrylamide). Western blotting was performed as described above.

## Data Availability

No large-scale data sets were generated or analyzed during this study. All data supporting the findings of this study are available from the corresponding author upon reasonable request.

## SUPPLEMENTAL MATERIAL

Table S1 aem.00326-25-s0001.pdf Strains, plasmids, and primers used in this study.

Table S2 aem.00326-25-s0002.pdf Viable cell counts after repeated cold stress (CFU/mL).

ASM does not own the copyrights to Supplemental Material that may be linked to, or accessed through, an article. The authors have granted ASM a non-exclusive, world-wide license to publish the Supplemental Material files. Please contact the corresponding author directly for reuse.
