# Supplementary material for: Replacement of branched-chain polyamine biosynthesis with thermospermine supports survival under both cold and heat stress in the hyperthermophilic archaeon Thermococcus kodakarensis
Source: Appl Environ Microbiol. 2025 May 28;91(6):e00326-25. doi: 10.1128/aem.00326-25 (PMC12175538; doi:10.1128/aem.00326-25)
Supplement: Table S1 — Strains, plasmids, and primers used in this study. [file aem.00326-25-s0001.pdf]

Table S1 Strains, plasmids and primers used in this study

Strain	Relevant characteristic(s) or sequence (5'–3')	Source or reference
E. coli		
DH5α	F^- Φ80d*lacZ*DM15 Δ(*lacZYA-argF*)U169 *deoR recA*1 *endA*1 *hsdR*17(rK^-, mK^+) *phoA supE*44 k- *thi*-1 *gyrA*96 *relA*	Stratagene
BL21-CodonPlus(DE3)-RIL	*E. coli* B F- *ompT hsdS*(rB^-mB^-) dcm^+ Tet^r *gal* λ(DE3) *endA,* Hte [*argU ileY leuW* Cam^r]	Agilent technologies
T. kodakaraensis		
KU216	Δ*pyrF*	Sato et al., Appl.Environ.Microbiol (2005)
DBP1	Δ*bpsA*:: *pdaD* Δ*pyrF*	Okada et al, J Bacteriol (2014)
KPS	Δ*bpsA*:: *speE* Δ*pyrF*	This study
Plasmid		
pET21a-Pc-SpeE	Expression plasmid for Pc-SpeE	Fukuda et al., Catalyst (2002)
pUD2-TK1691	pUD2 derivative harboring *bpsA* region along with its 5'- and 3'-flanking regions (ca. 1,000 bp each)	Okada et al, J Bacteriol (2014)
pUD2-Pc-speE	Replacing plasmid	This study
pTRC1	Parental plasmid for in vitro transcription	Endoh et al., 2006
pTRC11	Plasmid for in vitro transcription of *hyhL*	This study
Primers		

Ins PcSpeE Tk1691 updown Inf Fw	CTGGAGGTGTGAGATATGCGCAAGGTGCCCGGTCC	This study
Ins PcSpeE Tk1691 updown inf Rv	ATAAATCAGAAAGGCTCATCTGAGTTTTCTGTGTAT	This study
Revec Tk1691 updown Inf Fw	GCCTTTCTGATTTATTTTAAATTTAAAAAAG	This study
Revec Tk1691 updown Inf Rv	ATCTCACACCTCCAGAAGGATTC	This study
PespeE_seq_Fw	AAGGTTGTACTCTAGGC	This study
PespeE_seq_Md	TCAGAGAGGCGTTGAAAC	This study
PespeE_seq_Rv	AGAGAGCCCGTGGAAGAA	This study
F_pUD2-Pc-speE	GAATCCTTCTGGAGGTGTGA	This study
R_pUD2-Pc-speE	TGAGAACAGGAAGAAGAGATG	This study
F_bpsA	ATGAGGGAGATAATTGAGAGG	This study
F_speE	ATGCGCAAGGTGCCCGGTCCCATA	This study
hyhL_Fw	GAGCGGATAACAATTCCCCTCTAGACTACGACGTTGCCTGGT	This study
hyhL_Rv	ACAGCTATGACCATGATTACGAATTCATGCCCTCACCACTGGA	This study
pTRC11 _Fw	GAATTCGTAATCATGGTCATAGCTG	This study
pTRC11 _Rv	TCTAGAGGGGAATTGTTATCCGCTCACAATTCCCCTA	This study
