# Supplementary material for: Replacement of branched-chain polyamine biosynthesis with thermospermine supports survival under both cold and heat stress in the hyperthermophilic archaeon Thermococcus kodakarensis
Source: Appl Environ Microbiol. 2025 May 28;91(6):e00326-25. doi: 10.1128/aem.00326-25 (PMC12175538; doi:10.1128/aem.00326-25)
Supplement: Table S2 — Viable cell counts after repeated cold stress (CFU/mL). [file aem.00326-25-s0002.pdf]

Table S2. Viable cell counts after repeated cold stress (CFU/mL)

| Stress Cycle / Strains | 0 | 1 | 2 | 3 | 4 |
|---|---|---|---|---|---|
| KU216 | $1.21\times10^4$ | $1.19\times10^4$ | $9.85\times10^3$ | $9.65\times10^3$ | $9.53\times10^3$ |
| DBP1 | $1.11\times10^4$ | $7.50\times10^3$ | $5.0\times10$ | $5.0\times10$ | $6.0\times10$ |
| KPS | $6.65\times10^3$ | $6.42\times10^3$ | $3.53\times10^3$ | $3.22\times10^2$ | $2.27\times10^2$ |
